# In Silico Identification of Dual-Action Compounds Targeting TLR2 and *Streptococcus mutans* Proteins for the Prevention of Early Childhood Caries

**DOI:** 10.3390/dj14050301

**Published:** 2026-05-14

**Authors:** Juan Manuel Guzmán-Flores, Sofía Meza-Rodríguez, Sonia Isela Vázquez-Jiménez, Isabel del Carmen Medrano-González, Brianna Lissete Gallegos-García, Andrea Larissa Hernández-Villalobos, María Fernanda Yañez-Acosta, Carmen Celina Alonso-Sanchez

**Affiliations:** 1 Departamento de Ciencias de la Salud, Centro Universitario de los Altos, Universidad de Guadalajara, Tepatitlan de Morelos 47620, Jalisco, Mexico; sofia.meza9335@alumnos.udg.mx (S.M.-R.); sonia.vazquez@alumnos.udg.mx (S.I.V.-J.); brialisgal@gmail.com (B.L.G.-G.); larissand.nutri@gmail.com (A.L.H.-V.); 2 Departamento de Clínicas, Centro Universitario de los Altos, Universidad de Guadalajara, Tepatitlan de Morelos 47620, Jalisco, Mexico; mariaf.yaneza@academicos.udg.mx (M.F.Y.-A.); carmen.alonso@cualtos.udg.mx (C.C.A.-S.)

**Keywords:** early childhood caries, *Streptococcus mutans*, virtual screening, molecular docking, TLR2, bioinformatics, drug design, ADMET profiling

## Abstract

**Background/Objectives:** Early childhood caries (ECC) remains a major public health concern, with *Streptococcus mutans* as a primary etiological agent. Current treatments rely on broad-spectrum antimicrobials, which can disrupt the oral microbiome and promote resistance. This study applied a structure-based in silico pipeline to identify molecule modulators of Toll-like receptor 2 (TLR2), a key host receptor implicated in ECC, and to explore their binding potential against major *S. mutans* proteins. **Methods:** ECC-related genes were collected from public databases and analyzed by functional enrichment and protein–protein interaction (PPI) network analysis. Hub genes were ranked using centrality algorithms. Virtual screening on TLR2 (DrugCLIP) was followed by molecular docking of selected compounds against the TLR1/TLR2 heterodimer and 50 *S. mutans* proteins, complemented by in silico absorption, distribution, metabolism, excretion, and toxicity (ADMET) profiling. **Results:** Fifty-four ECC-related genes and nine hub genes were identified, with TLR2 and cathelicidin antimicrobial peptide (CAMP) as central nodes. Virtual screening yielded five lead compounds fulfilling drug-likeness and toxicity criteria. Docking to TLR1/TLR2 showed favorable binding energies, with Z7684613096 showing the most consistent binding. V026-2549 displayed the highest number of strong interactions with *S. mutans* targets, including dTDP-glucose 4,6-dehydratase (rmlB), NADP-dependent glyceraldehyde-3-phosphate dehydrogenase (gapN), glucosyltransferase C (gtfC), and 5-methyltetrahydropteroyltriglutamate-homocysteine methyltransferase (metE). **Conclusions:** Five candidate compounds with promising dual activity against TLR1/TLR2 and *S. mutans* proteins were prioritized for experimental validation, including TLR2 functional assays and in vitro anti-biofilm studies.

## 1. Introduction

Early childhood caries (ECC) is a prevalent multifactorial chronic disease affecting children worldwide, exerting a substantial burden on families, communities, and health care systems. ECC is defined as the presence of one or more decayed, missing, or filled primary tooth surfaces in children under six years of age. It progresses rapidly, frequently involving smooth surfaces, and adversely affects nutrition, growth, speech development, and overall quality of life [1]. A recent systematic review estimates a global pooled prevalence of approximately 48–49%, with considerable regional variation [2]. Alarmingly, temporal trend analyses covering 204 countries from 2010 to 2021 have revealed an upward trajectory, with the most pronounced increases observed in Latin America and the Caribbean and in high-income nations [3]. In Mexico, prevalence estimates remain particularly elevated, and behavioral and sociodemographic disparities continue to drive inequities in caries distribution among children [4,5]. These epidemiological patterns underscore the pressing need for novel, targeted therapeutic strategies that go beyond conventional preventive approaches.

*Streptococcus mutans* is widely recognized as a primary causative agent of dental caries, including ECC. This Gram-positive, facultatively anaerobic bacterium possesses a repertoire of virulence factors that enable it to colonize tooth surfaces and establish cariogenic biofilms [6]. Central among these are the glucosyltransferases (GtfB, GtfC, and GtfD), which synthesize extracellular glucans from dietary sucrose, providing the structural scaffold for biofilm adhesion and maturation [7,8]. Acid production from fermentable carbohydrates and acid-tolerance mechanisms further enable *S. mutans* to thrive at low pH, creating microenvironments hostile to competing commensal organisms [6]. Additional virulence determinants, including cell-surface adhesins of the Antigen I/II family (SpaP/Pac), glucan-binding proteins (GbpB, GbpC), two-component regulatory systems (VicRK, LiaSR), and dextranases, collectively orchestrate a sophisticated cariogenic machinery [9].

On the host side, the innate immune system plays a critical role in recognizing and counteracting *S. mutans* colonization. Toll-like receptor 2 (TLR2) is a key pattern-recognition receptor for Gram-positive pathogen-associated molecular patterns and is upregulated in response to *S. mutans* infection, triggering downstream inflammatory cascades through NF-κB activation [10]. TLR2 signaling operates on a threshold; basal activation is required for pathogen surveillance, but its chronic overstimulation by *S. mutans* lipoproteins in active carious lesions sustains a tissue-destructive inflammatory state that accelerates enamel demineralization without conferring additional protective benefit. The therapeutic goal, therefore, is not to broadly silence TLR2, but to attenuate its excessive activation at the local oral level [10]. Topical antagonism of Toll-like receptors has already been explored in periodontal research to curb tissue-destructive inflammation without impairing systemic defenses, a precedent directly relevant here. Delivery as a varnish, gel, or mouth rinse would confine the pharmacological effect to the oral cavity, preserving systemic innate immune competence, a consideration of particular weight in children under six years of age [11]. It must be acknowledged, however, that the functional classification of TLR2-binding compounds as agonists, antagonists, or partial modulators cannot be resolved computationally. Agonism would further amplify the inflammatory state we aim to attenuate, while antagonism or partial modulation would attenuate it. This distinction is central to the therapeutic rationale and will require cell-based validation as the immediate next step following this computational screen. The interplay between bacterial virulence and host immunity is a pivotal axis in ECC pathogenesis that remains underexplored therapeutically.

Current management of ECC relies predominantly on preventive measures, fluoride application, dietary counseling, and mechanical biofilm removal, complemented by restorative treatment once cavitation has occurred [1]. Broad-spectrum antimicrobials such as chlorhexidine have demonstrated efficacy in reducing *S. mutans* colonization; however, growing evidence indicates that prolonged chlorhexidine exposure may select for resistant bacterial populations, promote caries-associated microbial shifts, and induce cross-resistance to clinically important antibiotics [12]. Moreover, these agents indiscriminately disrupt the oral microbiome, potentially compromising the symbiotic balance essential for oral health [13]. This scenario has fueled interest in precision anti-virulence strategies that selectively target pathogenic mechanisms, such as glucosyltransferase-mediated biofilm formation, while preserving the commensal microbiota [14]. Previous work by our group has contributed to this evolving landscape by characterizing cytokine alterations in the saliva of children with caries and obesity [15], integrating proteomic data from salivary samples of children with caries through bioinformatic analysis [16], and employing network pharmacology and molecular docking to elucidate the molecular mechanisms of curcumin against dental caries and *S. mutans* [17].

In silico drug discovery has fundamentally transformed the identification of bioactive compounds by enabling rapid, cost-effective screening of vast chemical libraries against defined molecular targets [18]. Structure-based approaches, including virtual screening, molecular docking, and molecular dynamics simulations, enable researchers to predict binding modes, estimate affinities, and prioritize lead candidates with favorable pharmacokinetic and safety profiles before experimental validation [19]. Computational ADMET profiling further refines this pipeline by filtering compounds for drug-likeness, toxicity, and metabolic stability at an early stage [18,19]. Among recent innovations, DrugCLIP, a contrastive learning framework that embeds protein pockets and small molecules into a shared latent space, has demonstrated the ability to perform virtual screening up to 10 million times faster than conventional docking while maintaining competitive accuracy, opening new horizons for genome-scale drug discovery [20]. Concurrently, network pharmacology has emerged as a powerful systems-level strategy that integrates gene–disease associations, protein–protein interaction networks, and pathway enrichment analyses to identify key therapeutic targets within complex disease frameworks [17]. Despite these technological advances, the application of integrated in silico pipelines to identify dual-action compounds that simultaneously modulate host immune receptors and inhibit bacterial virulence proteins in the context of ECC remains largely unexplored.

This study applied a comprehensive in silico drug design pipeline with two sequential objectives: first, to identify small molecules targeting TLR2, the primary host receptor implicated in ECC, through structure-based virtual screening; and second, to assess their binding potential against *S. mutans* virulence and metabolic proteins through an exploratory docking phase.

## 2. Materials and Methods

### 2.1. Gene Retrieval and Functional Enrichment Analysis

Non-redundant genes associated with early childhood caries were retrieved from GeneCards [21] and HERB [22] databases. We searched for “early childhood caries”. The gene set was analyzed using ShinyGO V0.85 [23]. GO terms for biological processes, cellular components, and molecular functions were identified, along with significantly enriched KEGG pathways. Enrichment significance with FDR < 0.05 was considered significant. The complete list of 54 ECC-associated genes is provided in Supplementary Table S1.

### 2.2. Protein–Protein Interaction Network Construction

A protein–protein interaction (PPI) network was constructed for ECC-associated genes using the STRING database (V12.0) with a confidence score of 0.400 [24]. It was expanded by adding 20 first-neighbor proteins for a broader context. Visualization and analysis were done with Cytoscape V3.10.4 [25]. Topological parameters like nodes, edges, average neighbors, diameter, radius, path length, clustering coefficient, density, heterogeneity, and centralization were calculated to assess the network’s connectivity and organization.

### 2.3. Hub Gene Identification and Module Detection

Hub genes in the PPI network were identified using the CytoHubba V0.1 [26] plugin in Cytoscape, with four centrality algorithms: MCC, Degree, EPC, and Closeness. The top 10 nodes from each were visualized with a Venn diagram, and genes ranked top across all four were deemed common hub genes. Densely connected modules within the PPI network were detected using MCODE V2.0.3 in Cytoscape [26]. Each module underwent Gene Ontology enrichment analysis to reveal its main biological functions.

### 2.4. Transcription Factor and MicroRNA Regulatory Network Construction

To characterize the regulatory landscape of the identified hub genes, a co-regulatory network involving transcription factors (TFs) and microRNAs was constructed. Predicted TFs and candidate microRNAs regulating these hub genes were obtained from NetworkAnalyst [27]. The network topology was analyzed to identify regulators that are shared by multiple hub genes.

### 2.5. Virtual Screening and ADMET Profiling

Virtual screening targeted TLR2 as the primary receptor because it has a high-resolution ligand-binding domain structure in the PDB, essential for reliable structure-based screening. Most *S. mutans* virulence proteins lack such resolved structures, so docking against *S. mutans* proteins was secondary, using AlphaFold structures. TLR2, a membrane receptor with well-characterized ligand-binding sites and high-quality 3D structures, was chosen because chronic activation by *S. mutans* lipoproteins sustains inflammation in ECC. The screening focused on compounds that reduce this signaling rather than broadly inhibit TLR2. CAMP was excluded because it encodes LL-37, which is less suitable for docking. Virtual screening was performed using the DrugCLIP platform against six commercially available compound libraries: ChemDiv, Enamine Screening Collection, Vitas-M, FCHGroup SC 202007, Princeton BioMolecular Research, and the mcule HTS Library. DrugCLIP ranked all candidates by contrastive similarity score between the encoded TLR2 binding pocket and each ligand embedding; results were filtered to the top 100 hits, a cutoff selected based on the observed score distribution, where similarity values drop sharply beyond the highest-ranked compounds. This pool was sufficient to capture structural diversity while keeping downstream manual curation tractable [20].

The pharmacokinetic features and drug-like qualities of the top 100 hits were evaluated using SwissADME [28]. The compounds were checked against several drug-likeness rules, including Lipinski’s Rule of Five, Ghose filter, Veber rule, Egan rule, and Muegge filter, with any rule violations documented. Toxicity assessments, such as LD_50_ values for acute oral toxicity and toxicity class categorizations, were conducted via ProTox-3.0 [29]. Only compounds that satisfied the drug-likeness criteria and demonstrated acceptable toxicity profiles were chosen for molecular docking experiments. The identifiers and structural descriptors of the five candidate compounds are provided in Supplementary Table S1.

### 2.6. Molecular Docking to TLR1/TLR2 Heterodimer

The 3D structure of the TLR1/TLR2 heterodimer was retrieved from the Protein Data Bank (PDB ID: 2Z7X). Protein preparation included removing water molecules, adding hydrogen atoms, assigning charges, and performing energy minimization. The binding site was defined using the original ligand’s binding coordinates, extending 5 Å around the ligand. The docking grid box was centered at x = −15, y = −20, z = 1 Å with dimensions of 28 × 27 × 24 Å, fully enclosing the canonical lipopeptide-binding site at the TLR1/TLR2 dimer interface.

The five shortlisted compounds were prepared for docking with OpenBabel V3.1.0 [30], which involved generating 3D coordinates, assigning protonation states at physiological pH, and performing energy minimization for 1000 steps. Molecular docking was carried out using AutoDock Vina V1.2.6 [31]. Each ligand underwent 20 independent docking runs to explore conformational space. Binding free energies (ΔG) were calculated for all poses, and their distribution across the runs was displayed with box-and-whisker plots. The most representative pose for each compound, either the one with the median ΔG or the most common binding mode, was chosen for detailed interaction analysis.

### 2.7. Molecular Docking to Streptococcus mutans Proteins

A set of 50 *S. mutans* proteins with verified protein-level evidence was compiled from the UniProt database [32], applying two selection criteria: (1) reviewed Swiss-Prot annotation status, and (2) protein-level experimental evidence. Proteins lacking an AlphaFold model, or whose mean pLDDT fell below 70, were excluded. The three highest-affinity targets identified in the screen, rmlB (P95780, pLDDT = 97.94), gapN (Q59931, pLDDT = 98.44), and metE (Q8CWX6, pLDDT = 94.81) all returned high-confidence structural models, supporting the reliability of the docking results for these targets. The corresponding UniProt IDs were identified, and their three-dimensional structures were obtained from the Alphafold. Each of the five compounds was docked with all 50 *S. mutans* proteins. The binding free energies (ΔG, kcal/mol) for each compound–protein interaction were recorded, and the lowest ΔG value across all targets was noted for each compound. A threshold of −7.0 kcal/mol was used to define strong binding; the percentage of targets exceeding this threshold was calculated for each compound. Proteins that interacted strongly (ΔG < −7.0 kcal/mol) with all five compounds were classified as shared high-affinity targets.

For each of the five compounds, the *S. mutans* protein targets with binding energies below −7.0 kcal/mol were analyzed for Gene Ontology enrichment. This analysis was conducted using ShinyGO 0.85 [23]. Results with an FDR < 0.05 were considered statistically significant.

## 3. Results

### 3.1. Functional Enrichment of Genes Associated with ECC

**Figure 1:**
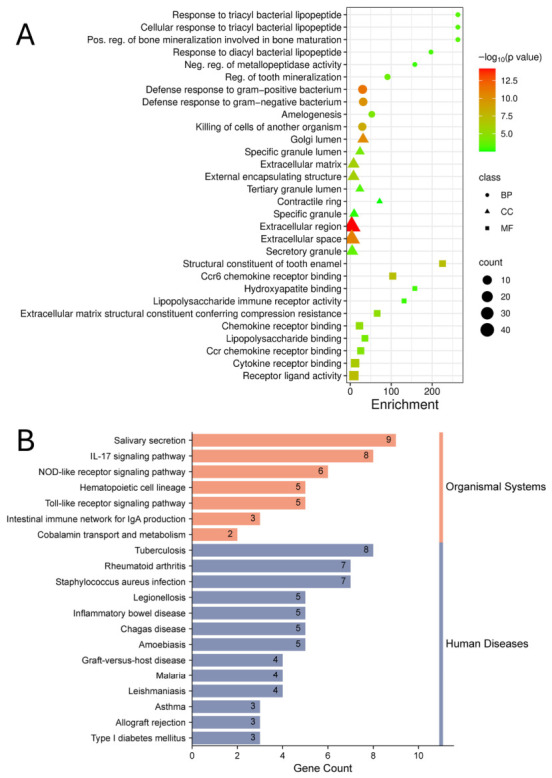
Genes associated with ECC and their functional enrichment. (**A**) Gene Ontology analysis showing significantly enriched terms for biological processes (BP), cellular components (CC), and molecular functions (MF). (**B**) Enriched KEGG signaling pathways for the same gene set.

A total of 54 non-redundant genes associated with ECC were retrieved from GeneCards and HERB and analyzed for function. Figure 1A shows Gene Ontology analysis indicating these genes mainly cluster in biological processes such as response to bacterial lipopeptides, defense against bacteria, regulation of tooth mineralization, and amelogenesis. Enriched cellular components and molecular functions include extracellular matrix, tooth enamel, secretory granules, luminal compartments, hydroxyapatite binding, chemokine binding, and lipopolysaccharide binding, reflecting their roles at the enamel mineral-immune response interface.

Figure 1B summarizes the significantly enriched KEGG pathways for the gene set. The top organismal system pathways include salivary secretion, IL-17 signaling, NOD-like receptor and Toll-like receptor signaling, and the intestinal immune network for IgA production, which are consistent with a mucosal immune response. Several genes map to human disease pathways, including tuberculosis, rheumatoid arthritis, *Staphylococcus aureus* infection, legionellosis, inflammatory bowel disease, and other infectious or immune-mediated conditions, suggesting that immune genes implicated in systemic inflammatory disorders may also influence susceptibility to ECC.

### 3.2. PPI Network of ECC-Related Proteins

**Figure 2:**
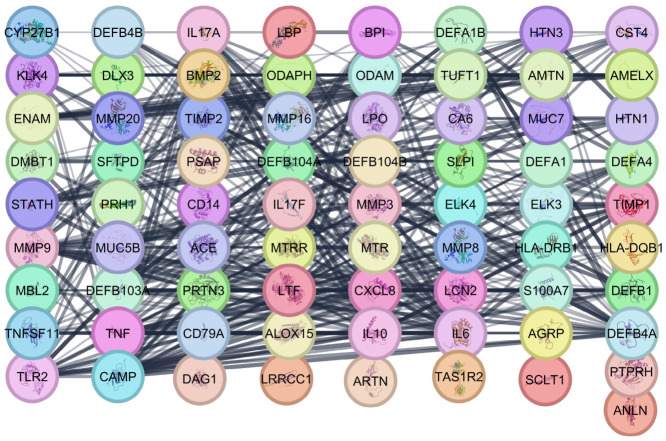
Protein–protein interaction (PPI) network of ECC-related genes. The network was constructed from the 54 genes associated with early childhood caries and extended by adding 20 first-neighbor proteins.

To analyze the connections among these ECC-associated genes, a protein–protein interaction (PPI) network was constructed and expanded by incorporating 20 first-neighbor proteins, yielding a total of 73 nodes and 434 edges (one gene was not identified). The network has an average of 13.152 neighbors per node, a diameter of 5, a radius of 3, and a mean path length of 2.169, indicating a densely connected, compact structure with short distances between proteins. Its global clustering coefficient of 0.647 and a network density of 0.202 indicate a strong tendency for proteins to form tightly knit groups. Additionally, the heterogeneity (0.684) and centralization (0.394) values point to several hub proteins that centralize interactions within the network (see Figure 2).

### 3.3. Identification of Hub Genes and Functional Modules in the ECC PPI Network

**Figure 3:**
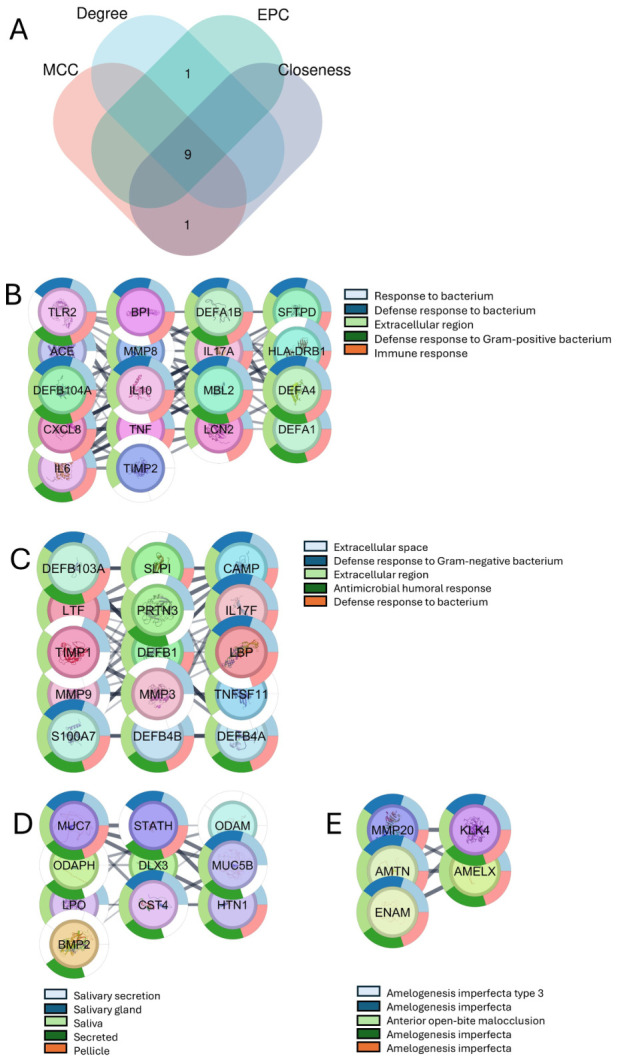
Hub genes and key modules in the ECC-related PPI network. (**A**) A Venn diagram highlights overlaps among the top 10 genes. (**B**–**E**) MCODE detects modules linked to antibacterial, immune, extracellular, salivary, and enamel formation functions, with TLR2 and CAMP as central nodes identified by CytoHubba and within MCODE clusters.

To identify hub genes in the ECC-related PPI network, a centrality analysis was conducted in CytoHubba using four algorithms: MCC, Degree, EPC, and Closeness. The top 10 nodes from each metric were visualized in a Venn diagram (Figure 3A). This identified nine common hub genes (CAMP, CXCL8, IL10, IL17A, IL6, LCN2, LTF, TLR2, and TNF) that consistently ranked high across all measures, indicating their key role in the ECC interaction network.

In parallel, MCODE was applied to the same PPI network to identify densely connected modules, yielding four relevant clusters (Figure 3B–E). The seed genes were TLR2 (module B), CAMP (module C), BMP2 (module D), and ENAM (module E). Each cluster was characterized by Gene Ontology enrichment, revealing themes related to antibacterial and immune responses (modules B and C), salivary secretion and acquired pellicle (module D), and enamel formation and amelogenesis-related disorders (module E). Notably, only TLR2 and CAMP, identified as central hubs by CytoHubba and as seed genes of MCODE modules, underscore their roles as key regulators of immune defense, salivary functions, and enamel biology in ECC.

### 3.4. Transcriptional and Post-Transcriptional Regulation of the Hub Genes

**Figure 4:**
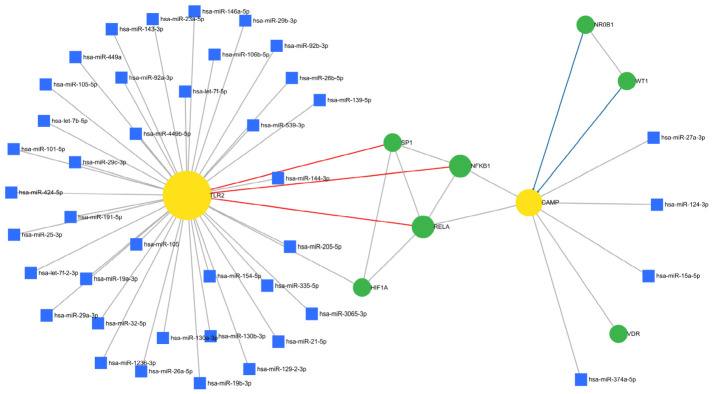
Predicted transcription factor and microRNA regulatory network for the hub genes TLR2 and CAMP. The network depicts TLR2 and CAMP as central hub genes (yellow circles), their TFs (green circles), and the microRNAs predicted to target each gene (blue squares).

A transcription factor (TF) and microRNA co-regulatory network was inferred for TLR2 and CAMP to characterize their regulatory landscape. Both hubs are central, surrounded by predicted upstream TFs and microRNAs, indicating dense control layers (Figure 4). TLR2 interacts with many microRNAs and TFs, such as SP1, HIF1A, NFKB1, and RELA, whereas CAMP has fewer, specific regulators, such as NR0B1, WT1, VDR, and microRNAs. Notably, NFKB1 and RELA are common TFs that regulate both and form an NF-κB–centered axis, suggesting that inflammatory and innate immune pathways co-modulate TLR2 and CAMP, linking pattern recognition and antimicrobial response in ECC.

### 3.5. Virtual Screening and ADMET/Toxicity Profiling of TLR2-Targeted Compounds

After virtual screening of TLR2 with DrugCLIP, the top 100 hits underwent pharmacokinetic filtering via SwissADME and toxicity prediction with ProTox-3. The five best compounds (Z9094100592, Z7684613096, Z9094100407, V026-2345, V026-2549) showed favorable drug-likeness, with low violations of Lipinski, Ghose, Veber, Egan, and Muegge rules, indicating acceptable physicochemical properties for oral bioavailability. All five compounds share a molecular weight at or below 521 Da, consensus LogP values between 3.19 and 4.48, high predicted GI absorption, and a bioavailability score of 0.55, indicating a consistent and favorable pharmacokinetic profile across the series. (Table 1 and Table 2).

Toxicity predictions placed all candidates within toxicity classes 3–6, with estimated LD50 values ranging from 300 to 7000 mg/kg, suggesting an overall low to moderate acute toxicity risk. Among them, Z7684613096 displayed the highest predicted toxicity (class 3, LD50 300 mg/kg), whereas V026-2549 showed the most benign profile (class 6, LD50 7000 mg/kg), making it particularly attractive for further optimization as a potential TLR2-modulating agent.

### 3.6. Molecular Docking of Compounds with the TLR1/TLR2 Heterodimer

**Figure 5:**
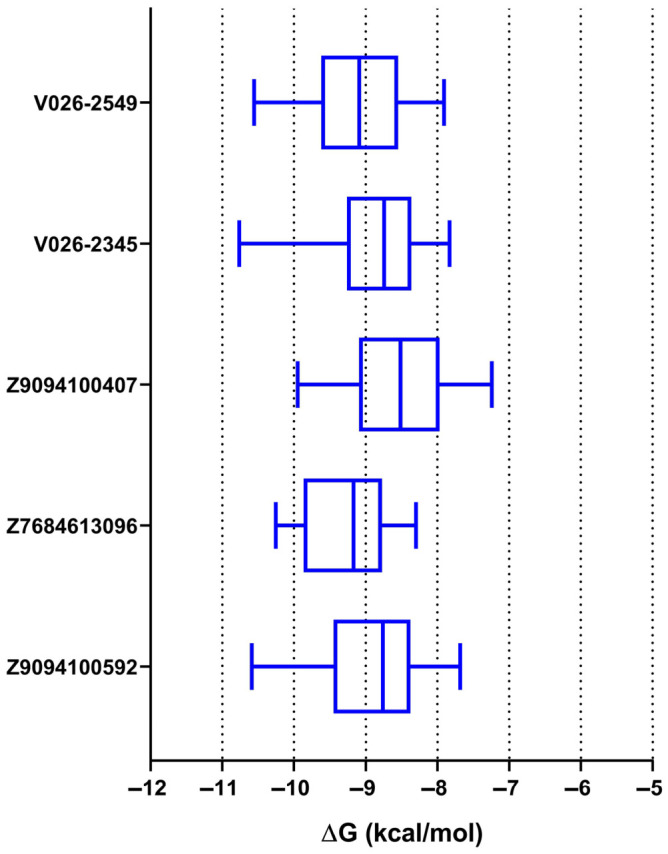
Distribution of docking binding free energies (ΔG) for five compounds bound to TLR1/TLR2. Each box-and-whisker plot shows 20 docking poses per ligand against the TLR1/TLR2 dimer, shown for ΔG values in kcal/mol.

To characterize the binding of the five molecules to the innate immune receptor, a docking analysis was performed against TLR1/TLR2 (PDB ID: 2Z7X). Across the 20 independent docking runs, Z7684613096 returned the lowest median ΔG (−9.167 kcal/mol, IQR: −9.798 to −8.800), with the narrowest interquartile range among the five candidates, indicating a well-defined and reproducible binding mode within the TLR1/TLR2 pocket. V026-2549 showed a comparable median (−9.087 kcal/mol, IQR: −9.531 to −8.592), while V026-2345 and Z9094100592 were intermediate (−8.743 and −8.760 kcal/mol, respectively). Z9094100407 returned the highest median ΔG (−8.512 kcal/mol, IQR: −9.051 to −8.135) and the widest overall range (−9.947 to −7.238 kcal/mol), reflecting greater conformational variability across runs. All values were negative across all poses for each compound, confirming energetically favorable binding in every case (Figure 5). All showed consistently negative ΔG values within a narrow range, indicating spontaneous, energetically favorable interactions with TLR1/TLR2.

Of the five candidates, Z7684613096 showed the most consistent binding and the smallest spread, indicating a well-defined binding mode within the TLR1/TLR2 pocket. V026-2549 and V026-2345 approached −11.0 kcal/mol but exhibited broader distributions, indicating that only some poses were optimally positioned. Z9094100407 was intermediate, while Z9094100592 had the broadest range. Overall, Z7684613096 combines favorable energy with high pose reproducibility.

**Figure 6:**
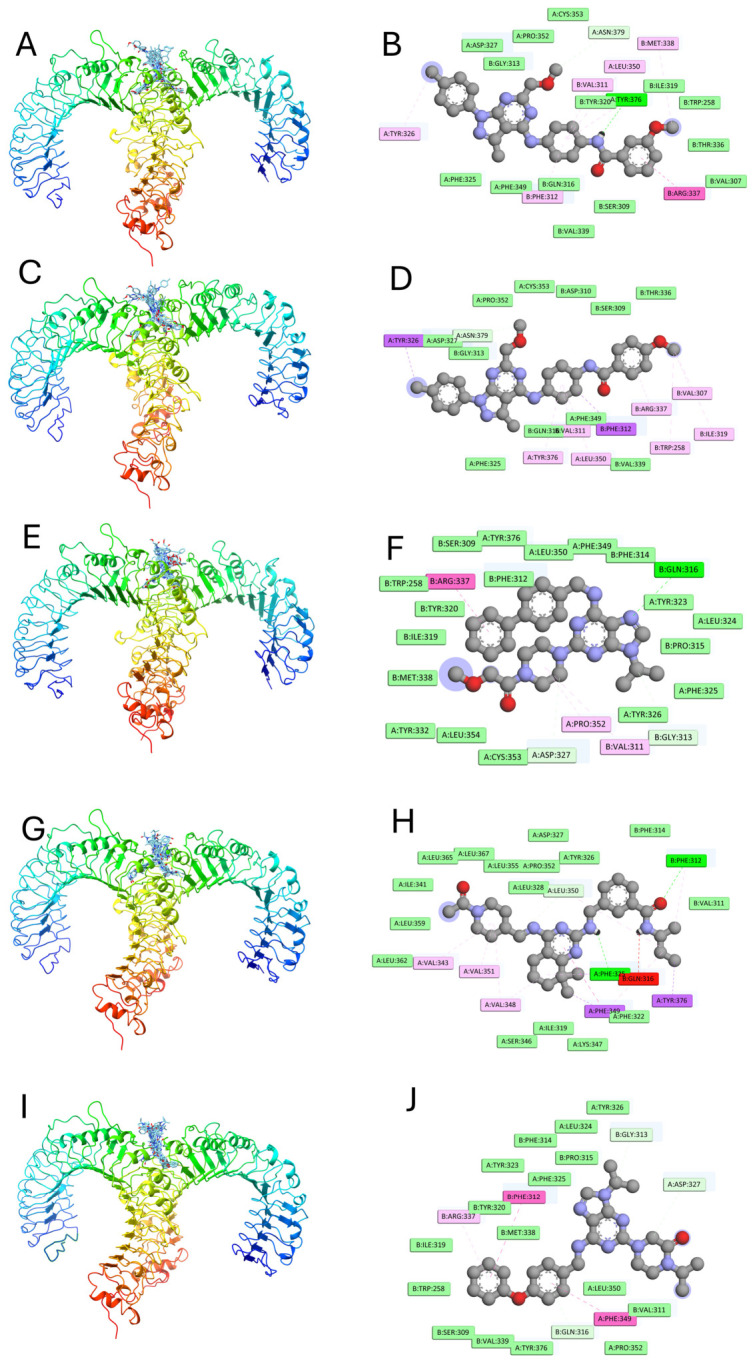
Three-dimensional binding modes and 2D interaction profiles of five TLR1/TLR2 ligands are shown. Panels (**A**,**B**) display V026-2549’s docking pose in the TLR1/TLR2 heterodimer and the 2D interaction map with hydrophobic contacts in purple and pink, and hydrophilic interactions in green. Panels (**C**–**J**) show the binding of V026-2345, Z9094100407, Z7684613096, and Z9094100592, respectively.

To understand how the five candidate molecules fit in the TLR1/TLR2 binding pocket, their key docking poses were visualized in 3D along with detailed 2D interaction maps (Figure 6). Each ligand occupies the canonical binding groove at the dimer interface, with the 2D diagrams clearly distinguishing hydrophobic (purple and lilac) from hydrophilic (green) contacts. All compounds share a core set of non-polar contacts and specific polar interactions, despite some variation, indicating a broadly similar interaction environment.

### 3.7. Molecular Docking of the Five Compounds Against Streptococcus mutans Proteins

Beyond TLR1/TLR2, we examined the direct effects of five compounds on *Streptococcus mutans* virulence and metabolism. We retrieved 50 reviewed proteins with protein-level evidence from UniProt and docked each against the five molecules.

Docking against all 50 *S. mutans* proteins was performed as blind docking, with the search space covering the full protein surface. AlphaFold-predicted structures were quality-filtered using a pLDDT > 70 threshold prior to interpretation; models falling below this cutoff were excluded from further analysis.

**Figure 7:**
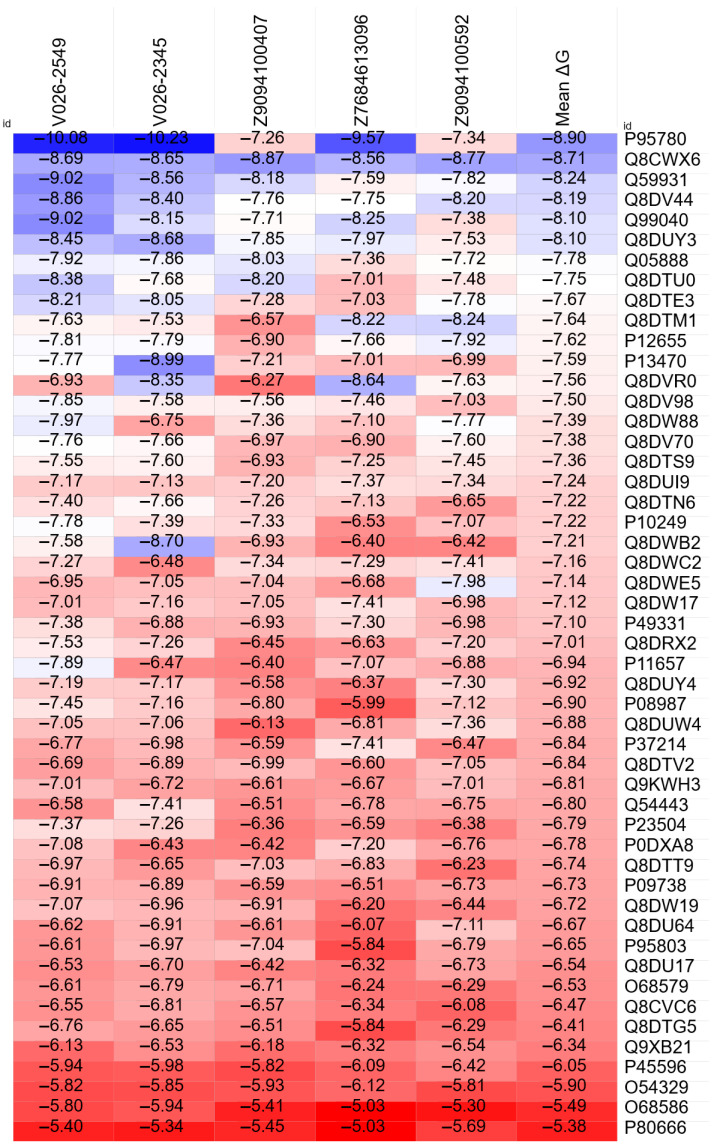
Heatmap of binding free energies (ΔG, kcal/mol) obtained by molecular docking against *S. mutans* protein targets across five ligands (V026-2549, V026-2345, Z9094100407, Z7684613096, Z9094100592). Proteins are ranked by mean ΔG (ascending). Bluer colours are indicative of negative values, while redder colours are indicative of positive values.

As Figure 7 illustrates, compounds V026-2549 and V026-2345 recorded the most favorable overall ΔG values, closely followed by Z9094100592, Z7684613096, and Z9094100407.

Across the 250 compound–protein pairs, the strongest interaction involved rmlB (P95780), an enzyme vital for *S. mutans* polysaccharides. As seen in the top row of the heatmap, V026-2345 returned the lowest predicted ΔG for this target (−10.23 kcal/mol), followed by V026-2549 and Z7684613096 at −10.08 and −9.57 kcal/mol, respectively. These values should be interpreted as relative rankings within this computational screen, not as experimental binding affinities.

Alongside rmlB, this select group includes other high-affinity targets essential for bacterial metabolism and virulence, such as metE (Q8CWX6), gapN (Q59931), and dexB (Q99040).

Applying a threshold of −7.0 kcal/mol to define strong binding, V026-2549 stands out: 32 of its 50 interactions (64%) surpass this cutoff. It is followed by V026-2345 (28; 56%), Z9094100592 (27; 54%), Z7684613096 (24; 48%), and Z9094100407 (20; 40%).

A revealing finding is that 11 of these *S. mutans* proteins (rmlB, dgkA, gapN, metE, cas9, deoB, fni, SMU_747c, argB, folT, and dexB) crossed the −7.0 kcal/mol barrier across all five evaluated compounds. This points to a common set of highly susceptible bacterial targets that regulate critical pathways, including polysaccharide biosynthesis, nucleotide salvage, and central carbon metabolism.

### 3.8. Gene Ontology Enrichment of High-Affinity S. mutans Protein Targets

**Figure 8:**
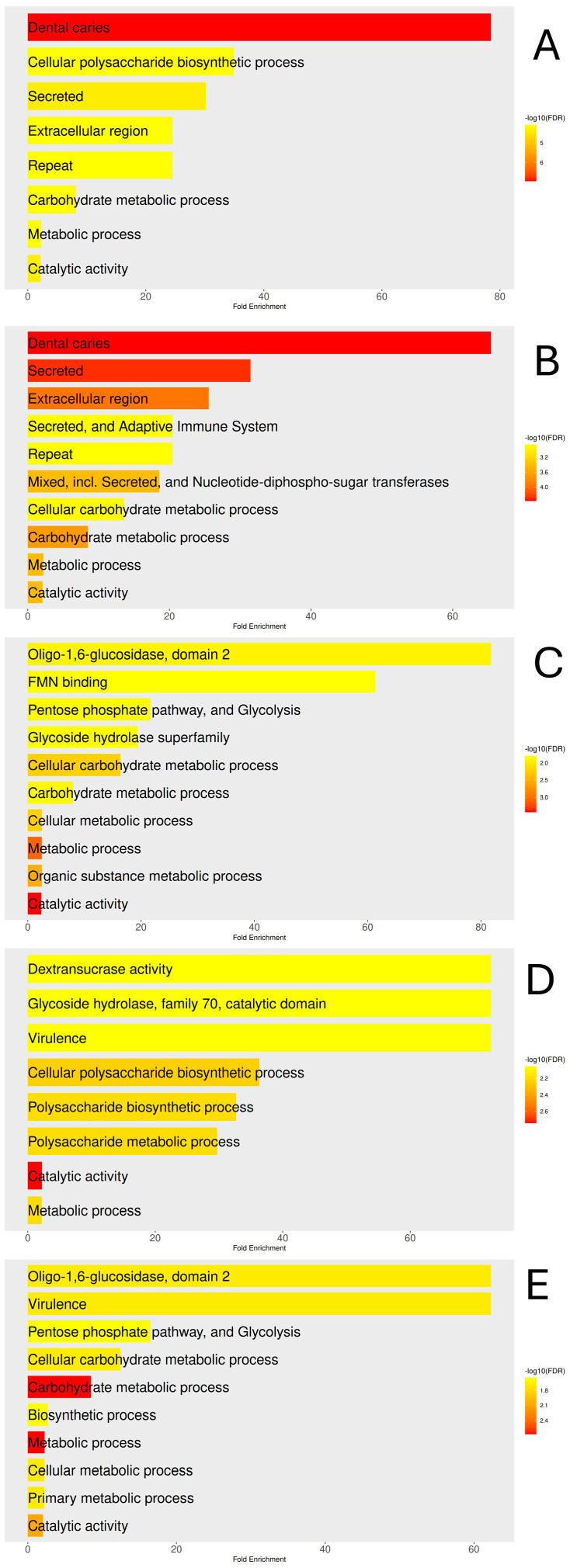
Gene Ontology enrichment analysis of *Streptococcus mutans* protein targets with binding energies below −7.0 kcal/mol for each candidate compound. Bars represent fold enrichment, and color intensity reflects the −log10(FDR), with warmer tones indicating higher statistical significance. (**A**) V026-2549; (**B**) V026-2345; (**C**) Z9094100407; (**D**) Z7684613096; (**E**) Z9094100592.

To understand *S. mutans* proteins that interacted best with each compound, a Gene Ontology analysis was performed on targets with binding energy below −7.0 kcal/mol (Figure 8). All five panels show enriched terms such as carbohydrate metabolic process, catalytic activity, and metabolic process, indicating that the molecules target proteins involved in central carbon metabolism and essential enzymatic functions for bacterial survival.

Panels A and B, representing V026-2549 and V026-2345, stand out as their top enriched term is “dental caries,” linking the predicted targets of these compounds to the disease. Both highlight cellular polysaccharide biosynthesis, secreted proteins, and the extracellular region, consistent with their affinity for virulence enzymes such as glucosyltransferases and rmlB. Panel B also shows terms related to the immune system and nucleotide-diphospho-sugar transferases, indicating a broader role for V026-2345.

Panels C and E (Z9094100407 and Z9094100592) show similar enrichment in oligo-1,6-glucosidase, FMN binding, and pathways such as the pentose phosphate and glycolysis, indicating an effect on intracellular sugar catabolism rather than on extracellular virulence factors. Panel D (Z7684613096) has a unique profile with dextransucrase activity, glycoside hydrolase family 70, and virulence, as well as polysaccharide biosynthesis and metabolism, highlighting its potential to disrupt glucan-mediated biofilm formation.

## 4. Discussion

Our study identifies promising anti-virulence candidates against *S. mutans* with good binding profiles and predicted drug-likeness, supporting their potential for targeted therapies to prevent or manage dental caries, though experimental validation is needed. Using network pharmacology, virtual screening, molecular docking, and ADMET profiling, we found five lead compounds active against the host immune receptor TLR2 and *S. mutans* proteins. This approach enabled us to select molecules with strong binding, favorable pharmacokinetics, and links to cariogenic pathways.

The analysis of 54 ECC-associated genes highlighted clusters in bacterial defense, tooth mineralization, and mucosal immunity. Toll-like receptor, IL-17, and NOD-like receptor pathways underscore the role of innate immunity in responding to cariogenic bacteria. The PPI network exhibited high connectivity and short paths, indicating efficient information flow and the presence of key regulatory nodes. Four centrality algorithms identified nine hub genes, with *TLR2* and *CAMP* being both central and pivotal as seed genes of MCODE modules. TLR2, a pattern recognition receptor on immune and epithelial cells, detects lipoproteins and lipoteichoic acids from Gram-positive bacteria like *S. mutans* [33]. Studies show increased salivary TLR2 in children with ECC, decreasing after treatment, indicating TLR2’s role in ECC [34]. In addition, the function of these molecules can be regulated by certain agonists [35]. CAMP encodes LL-37, an antimicrobial peptide that kills bacteria and modulates inflammation [36]. In a previous study, which also evaluated vitamin D, high salivary levels of LL-37 were observed in children with dental caries [37]. NFKB1 and RELA are transcription factors regulating TLR2 and CAMP, forming an NF-κB regulatory axis. This link suggests TLR2 as a therapeutic target to modulate immune and antimicrobial responses, relevant for high-risk pediatric patients.

This network-based target selection strategy aligns with the approach adopted by Guzmán-Flores et al. [17], who applied network pharmacology and molecular docking to elucidate the dual action of curcumin on both host carious processes and *S. mutans* metabolic pathways. Their study identified seven key proteins, MAPK1, BCL2, KRAS, CXCL8, TGFB1, MMP9, and IL1B, through which curcumin modulates inflammation and apoptosis in caries [17]. In a similar vein, Gómez-García et al. [38] demonstrated the expression of proinflammatory cytokines, including CXC8, in the dental pulp of carious teeth from Mexican individuals [38]. This finding further validates the biological relevance of immune-mediated hub genes in the pathogenesis of ECC.

The decision to target TLR2 rather than CAMP in virtual screening was informed by structural factors, as outlined below: TLR2 possesses a well-defined ligand-binding ectodomain that is amenable to docking. In contrast, LL-37 is a diminutive, adaptable, cationic peptide that poses significant challenges for small-molecule targeting. Following virtual screening with DrugCLIP, the top 100 hits were subjected to pharmacokinetic and toxicity filtering. Five compounds (Z9094100592, Z7684613096, Z9094100407, V026-2345, and V026-2549) successfully passed all filters, exhibiting minimal violations of drug-likeness criteria such as Lipinski, Ghose, Veber, Egan, and Muegge, suggesting promising oral bioavailability. The observed toxicity levels ranged from class 3 (Z7684613096, LD50 300 mg/kg) to class 6 (V026-2549, LD50 7000 mg/kg), with most candidates falling within acceptable safety margins. These results highlight the utility of integrating AI-driven virtual screening with classical pharmacokinetic filters to accelerate the early stages of anti-caries drug discovery, in agreement with recent reviews emphasizing the transformative role of computational ADMET profiling in modern drug development [14]. One ambiguity this study cannot resolve computationally is whether the five candidates act as TLR2 antagonists or partial agonists. Docking reveals favorable binding within the canonical lipopeptide pocket at the TLR1/TLR2 dimer interface, but binding affinity alone does not determine functional outcome. This distinction matters clinically; a compound that fully silences TLR2 would impair pathogen detection, while one that competitively attenuates overstimulation would align with the therapeutic objective of reducing caries-associated inflammation without compromising host defense. Resolving this requires a stepwise experimental approach; NF-κB luciferase reporter assays in HEK293 cells stably expressing TLR1/TLR2 would determine whether each compound activates or suppresses receptor signaling, and cytokine profiling, specifically IL-6, TNF-α, and IL-10 secretion from TLR2-stimulated macrophages exposed to each compound, would provide a functional immune readout. These assays constitute the most pressing next experimental step and will be addressed in a follow-up investigation.

All five compounds returned negative predicted binding free energies against the TLR1/TLR2 heterodimer, ranging from approximately −9.0 to −11.0 kcal/mol. While these values support prioritization of candidates for experimental follow-up, they are scoring-function estimates and should not be equated with experimentally determined dissociation constants. The −7.0 kcal/mol cutoff applied to *S. mutans* protein targets was selected based on the established benchmark performance of AutoDock Vina, in which values in this range are associated with moderate predicted affinity for drug-like molecules. This threshold was applied uniformly across all compounds–protein pairs to enable consistent relative ranking. As a reference point, small-molecule TLR2 inhibitors reported in the literature, including alpha-amyrin, show docking energies in a comparable range prior to experimental confirmation of activity. Our candidates fall within this bracket, which supports their selection for downstream validation while acknowledging that computational scores require experimental corroboration. Beyond scoring limitations, molecular docking captures a static estimate of binding likelihood and does not confirm biological activity, functional selectivity, or binding stability under physiological conditions. Protein flexibility, explicit solvation, entropic contributions, and induced-fit effects are not captured by the scoring function. Molecular dynamics simulations would address some of these limitations by sampling conformational space over time; their inclusion was beyond the scope of this initial screening study, but is planned for the experimental follow-up phase.

Z7684613096 exhibited the narrowest interquartile range across 20 independent docking runs, suggesting a well-defined and reproducible binding mode within the canonical lipopeptide pocket at the dimer interface. The process of TLR1/TLR2 heterodimerization is imperative for the recognition of triacylated lipoproteins derived from Gram-positive bacteria. Theoretically, the modulation of this receptor could attenuate excessive inflammatory signaling while maintaining basal immunosurveillance. A previous in silico and in vitro study identified alpha-amyrin as a potent TLR2 inhibitor for bacterial infection therapeutics, thereby supporting the feasibility of small-molecule modulation of this receptor [35]. However, the docking energies observed here should be interpreted with caution, since molecular docking provides snapshots and does not fully capture the dynamic nature of protein–ligand interactions or solvation effects.

V026-2549 showed the broadest predicted coverage across *S. mutans* proteins, with 64% of the 50 targets returning ΔG values below the −7.0 kcal/mol threshold used here as a relative filter for prioritization. This threshold is a computational criterion, not a validated predictor of in vitro activity, and its application across structurally diverse bacterial proteins carries inherent uncertainty. For V026-2549, the most robust interactions included rmlB (P95780), dexB (Q99040), gapN (Q59931), and argB (Q8DV44). Notably, gtfC (glucosyltransferase C, P13470) also demonstrated strong binding affinities, particularly with V026-2345 (−8.99 kcal/mol). It has been demonstrated that rmlB plays a pivotal role in the biosynthesis of dTDP-L-rhamnose. This sugar nucleotide is indispensable for the assembly of rhamnose-glucose polysaccharides (RGPs) in the *S. mutans* cell wall. Disruption of dTDP-L-rhamnose biosynthesis has been demonstrated to compromise streptococcal viability and virulence. Furthermore, inhibitory lead compounds targeting RmlB have exhibited activity against both streptococcal pathogens and *Mycobacterium tuberculosis*. Consequently, the high affinity of V026-2549 for rmlB suggests a potential mechanism by which it could compromise bacterial cell wall integrity and, in turn, affect bacterial fitness [39,40,41].

The strong binding to gtfC is equally significant. Glucosyltransferases (GtfB, GtfC, GtfD) synthesize extracellular glucans that constitute the structural scaffold of cariogenic biofilms. Inhibition of these enzymes is one of the most actively pursued anti-virulence strategies in caries research. Recently, molecular insights into GTF inhibitors were provided using density functional theory, molecular docking, and molecular dynamics simulations, confirming that compounds with binding energies exceeding those of acarbose can significantly disrupt biofilm architecture. The observation that V026-2549 and V026-2345 also enriched Gene Ontology terms directly linked to “dental caries” reinforces their specificity for cariogenic mechanisms and distinguishes them from compounds targeting solely intracellular metabolic enzymes [8,42,43].

The computational analysis elucidated ten high-affinity targets shared across the five evaluated compounds. These top-ranked candidates, specifically rmlB (P95780), metE (Q8CWX6), gapN (Q59931), argB (Q8DV44), dexB (Q99040), SMU_747c (Q8DUY3), dgkA (Q05888), deoB (Q8DTU0), cas9 (Q8DTE3), and aspB (Q8DTM1), underscore a conserved vulnerability in *Streptococcus mutans* that spans polysaccharide biosynthesis, central carbon metabolism, and nucleotide salvage pathways. This multi-target binding profile aligns with the concept of polypharmacology, which is increasingly embraced in network pharmacology approaches, suggesting that these compounds may exert their antimicrobial effects through a synergistic disruption of essential metabolic networks rather than a single-target mechanism [14].

Regarding the TLR2 component specifically, the study does not establish whether the candidates act as agonists or antagonists; docking scores reflect binding affinity rather than signaling outcome. The underlying premise, that attenuating chronic TLR2-driven inflammation at the caries site could be therapeutically useful, is biologically grounded but unverified. Confirming it will require NF-κB activation assays, cytokine profiling in oral epithelial models, and selectivity testing across the TLR family. The pediatric context adds another layer; any compound modulating innate immune receptors in children under six must clear a higher safety bar, and that evaluation belongs in dedicated in vivo models, not in silico predictions.

It is imperative to acknowledge the limitations inherent in this approach. Initially, the results are derived from computational predictions and lack empirical validation. Molecular docking provides estimates of binding energy; however, it does not account for protein flexibility, entropic effects, or the oral cavity environment. AlphaFold-predicted structures carry inherent uncertainty in docking applications, with published benchmarks reporting a mean auROC of ~0.48 compared to ~0.60 for experimental structures. Blind docking was used to avoid bias in binding site selection, and a pLDDT > 70 filter was applied to retain only structurally reliable models. A conservative −7.0 kcal/mol threshold was additionally used to define strong binding and reduce false positives. The top-ranked targets, rmlB, gapN, and metE, all exceeded pLDDT > 94, supporting greater confidence in those specific predictions. Results for lower-confidence targets should be treated as directional until experimental structures become available [44,45,46]. CAMP was excluded due to challenges experienced with docking small, cationic, flexible peptides. Future work should explore molecular dynamics or peptide–ligand modeling to assess effects on LL-37. ADMET profiling is employed exclusively in silico via tools such as SwissADME and ProTox-3.0. However, these tools exhibit varying levels of accuracy, necessitating experimental validation before clinical implementation [47,48]. Consequently, in vitro tests against *S. mutans* biofilms and oral commensals, in conjunction with in vivo studies on caries models, are imperative to substantiate anti-virulence activity, efficacy, bioavailability, and safety.

These findings are strictly computational and represent an early-stage prioritization of candidates. The identification of compounds with predicted dual activity, binding the TLR1/TLR2 interface and showing favorable docking profiles against *S. mutans* proteins, supports the rationale for precision anti-infective strategies that could, in principle, spare the oral microbiome. However, whether this selectivity holds in biological systems remains to be demonstrated. The drug-likeness and toxicity profiles of the five candidates, particularly V026-2549, are consistent with topical oral delivery formats such as varnishes or gels, but pediatric suitability cannot be established from computational data alone. Any conclusions regarding clinical efficacy, microbiome safety, or applicability to other oral diseases, including periodontitis or peri-implantitis, require experimental validation as a prerequisite. The integration of AI-driven virtual screening with network pharmacology does provide a scalable framework applicable beyond ECC, but that potential should be evaluated in future studies rather than assumed from the present results.

The present study is intentionally scoped to the computational phase of a broader drug discovery effort. Experimental validation, including NF-κB reporter assays in TLR2-expressing macrophages to determine agonist versus antagonist activity, in vitro minimum inhibitory concentration assays against *S. mutans*, and biofilm inhibition studies, constitutes the direct next step and will be addressed in a follow-up investigation. This staged approach is consistent with established in silico-to-in vitro pipelines in the field and allows the computational methodology to be reported and scrutinized independently before experimental resources are committed to the most promising candidates.

## 5. Conclusions

This study applied an integrated in silico pipeline, combining network pharmacology, AI-driven virtual screening, molecular docking, and ADMET profiling, to identify five candidate compounds with predicted dual binding activity against the TLR1/TLR2 heterodimer and *S. mutans* virulence and metabolic proteins. Network analysis of 54 ECC-associated genes identified *TLR2* and *CAMP* as central nodes, supporting TLR2 as a computationally justified target. Among the five candidates, Z7684613096 showed the most reproducible predicted binding mode at the TLR1/TLR2 interface, while V026-2549 returned the broadest coverage across *S. mutans* protein targets. Drug-likeness and toxicity profiles were consistent with topical oral delivery. These results are hypothesis-generating. They do not establish biological activity, clinical efficacy, or safety, all of which require experimental validation. The immediate next steps are cell-based TLR2 functional assays, cytokine profiling, MIC determination against *S. mutans*, and in vitro biofilm inhibition studies. The five candidates prioritized here provide a defined starting point for that experimental phase.

## Figures and Tables

**Table 1:** Chemical structures of the five TLR2-targeting compounds. Molecular weight (MW), consensus LogP, gastrointestinal absorption, and oral bioavailability score, as calculated by SwissADME, are annotated below each structure.

Molecule	Formula	MW	Consensus LogP	GI Absorption	Bioavailability Score	
Z9094100592	C28H33N7O2	499.61	3.76	High	0.55	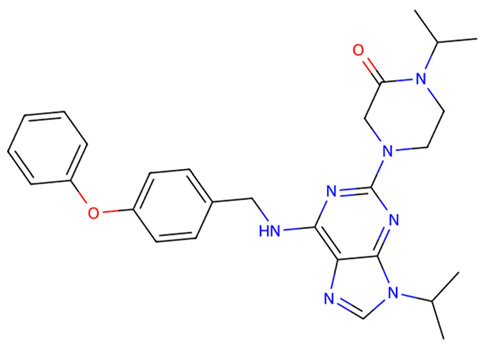
Z7684613096	C30H44N6O2	520.71	4.48	High	0.55	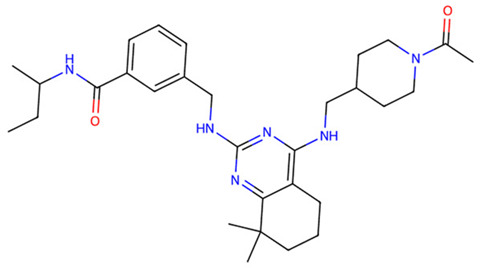
Z9094100407	C28H33N7O2	499.61	3.19	High	0.55	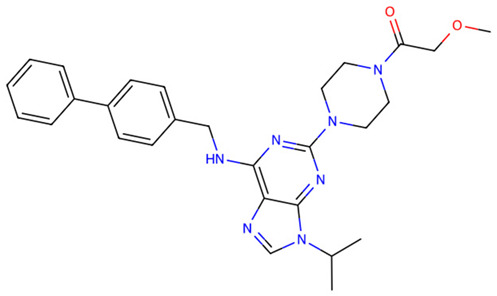
V026-2345	C29H34N6O3	514.62	4.11	High	0.55	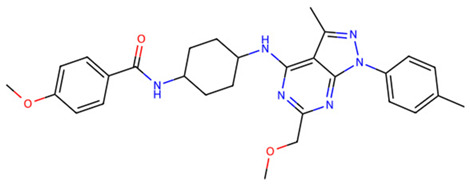
V026-2549	C29H34N6O3	514.62	4.12	High	0.55	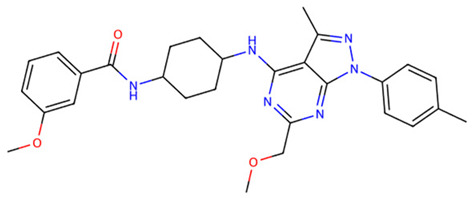

**Table 2:** Drug-likeness and toxicity profile of TLR2-targeting compounds.

Molecule	Lipinski *	Ghose *	Veber *	Egan *	Muegge *	LD50 (mg/kg)	Toxicity Class
**Z9094100592**	0	2	0	0	0	500	4
**Z7684613096**	1	3	1	0	1	300	3
**Z9094100407**	0	2	0	0	0	1640	4
**V026-2345**	1	3	0	0	0	1600	4
**V026-2549**	1	3	0	0	0	7000	6

* Number of violations.

## Data Availability

Data are contained within the article.

## Supplementary Materials

The following supporting information can be downloaded at: https://www.mdpi.com/article/10.3390/dj14050301/s1, Table S1: manuscript-supplementary.xlsx.

## References

[1] Zou J., Du Q., Ge L., Wang J., Wang X., Li Y., Song G., Zhao W., Chen X., Jiang B. (2022). Expert consensus on early childhood caries management. Int. J. Oral Sci..

[2] Uribe S.E., Innes N., Maldupa I. (2021). The global prevalence of early childhood caries: A systematic review with meta-analysis using the WHO diagnostic criteria. Int. J. Paediatr. Dent..

[3] Costa C.M., Alves-Costa S., Hugo F.N., Paes A.M.A., Tabchoury C.P.M., Cury J.A., Ribeiro C.C.C. (2025). Increasing Global Trends in Early Childhood Caries Prevalence over the Last Decade: Global Burden of Disease Study 2021. Caries Res..

[4] Marquez-Perez K., Zuniga-Lopez C.M., Torres-Rosas R., Argueta-Figueroa L. (2023). Reported prevalence of dental caries in Mexican children and teenagers. Rev. Med. Inst. Mex. Seguro Soc..

[5] Perez-Reyes A., Becerra-Ruiz J.S., Guzman-Flores J.M. (2025). Influence of Behavioral and Sociodemographic Factors on Dental Caries in Mexican Children. Pediatr. Rep..

[6] Lemos J.A., Palmer S.R., Zeng L., Wen Z.T., Kajfasz J.K., Freires I.A., Abranches J., Brady L.J. (2019). The Biology of *Streptococcus mutans*. Microbiol. Spectr..

[7] Hashizume-Takizawa T., Ando T., Urakawa A., Aoki K., Senpuku H. (2025). Cell wall glycosyltransferase of *Streptococcus mutans* impacts its dissemination to murine organs. Infect. Immun..

[8] Zhang Q., Ma Q., Wang Y., Wu H., Zou J. (2021). Molecular mechanisms of inhibiting glucosyltransferases for biofilm formation in *Streptococcus mutans*. Int. J. Oral Sci..

[9] Fitri D.K., Tuygunov N., Wan Harun W.H.A., Purwasena I.A., Cahyanto A., Zakaria M.N. (2025). Key virulence genes associated with *Streptococcus mutans* biofilm formation: A systematic review. Front. Oral Health.

[10] Al-Alawi F.Z.M., Kariminik A., Tajbakhsh E. (2024). Toll-like receptors and *Streptococcus mutans*: An updated review article. Allergol. Immunopathol..

[11] Gao S., Li H., Li Z., Wang H., Li X., Yang S., Huang L., Zhang B., Zhang K., Tsoi J.K.H. (2025). Multifunctional Injectable Bioadhesive with Toll-like Receptor 4 and Myeloid Differentiation Factor 2 Antagonistic Anti-inflammatory Potential for Periodontal Regeneration. ACS Nano.

[12] Fruh R., Anderson A., Cieplik F., Hellwig E., Wittmer A., Vach K., Al-Ahmad A. (2022). Antibiotic Resistance of Selected Bacteria after Treatment of the Supragingival Biofilm with Subinhibitory Chlorhexidine Concentrations. Antibiotics.

[13] Cieplik F., Jakubovics N.S., Buchalla W., Maisch T., Hellwig E., Al-Ahmad A. (2019). Resistance Toward Chlorhexidine in Oral Bacteria—Is There Cause for Concern?. Front. Microbiol..

[14] Chen Z., Zhao X., Zheng H., Wang Y., Zhang L. (2025). Advances and challenges in drug design against dental caries: Application of in silico approaches. J. Pharm. Anal..

[15] Ramirez-De Los Santos S., Lopez-Pulido E.I., Medrano-Gonzalez I.D.C., Becerra-Ruiz J.S., Alonso-Sanchez C.C., Vazquez-Jimenez S.I., Guerrero-Velazquez C., Guzman-Flores J.M. (2021). Alteration of cytokines in saliva of children with caries and obesity. Odontology.

[16] Juan Manuel G.-F., Fernando M.-E., Julieta Sarai B.-R., Sandra Berenice V.-R. (2023). Integration of Proteomic Data Obtained from the Saliva of Children with Caries through Bioinformatic Analysis. Curr. Proteom..

[17] Guzman-Flores J.M., Perez-Reyes A., Vazquez-Jimenez S.I., Isiordia-Espinoza M.A., Martinez-Esquivias F. (2024). A Docking and Network Pharmacology Study on the Molecular Mechanisms of Curcumin in Dental Caries and *Streptococcus mutans*. Dent. J..

[18] Shoichet B.K. (2004). Virtual screening of chemical libraries. Nature.

[19] Wu T., Jiang W. (2025). Computational-aided drug design strategies for drug discovery and development against oral diseases. Front. Pharmacol..

[20] Jia Y., Gao B., Tan J., Zheng J., Hong X., Zhu W., Tan H., Xiao Y., Tan L., Cai H. (2026). Deep contrastive learning enables genome-wide virtual screening. Science.

[21] Stelzer G., Rosen N., Plaschkes I., Zimmerman S., Twik M., Fishilevich S., Stein T.I., Nudel R., Lieder I., Mazor Y. (2016). The GeneCards Suite: From Gene Data Mining to Disease Genome Sequence Analyses. Curr. Protoc. Bioinform..

[22] Fang S., Dong L., Liu L., Guo J., Zhao L., Zhang J., Bu D., Liu X., Huo P., Cao W. (2021). HERB: A high-throughput experiment- and reference-guided database of traditional Chinese medicine. Nucleic Acids Res..

[23] Ge S.X., Jung D., Yao R. (2020). ShinyGO: A graphical gene-set enrichment tool for animals and plants. Bioinformatics.

[24] Szklarczyk D., Kirsch R., Koutrouli M., Nastou K., Mehryary F., Hachilif R., Gable A.L., Fang T., Doncheva N.T., Pyysalo S. (2023). The STRING database in 2023: Protein-protein association networks and functional enrichment analyses for any sequenced genome of interest. Nucleic Acids Res..

[25] Shannon P., Markiel A., Ozier O., Baliga N.S., Wang J.T., Ramage D., Amin N., Schwikowski B., Ideker T. (2003). Cytoscape: A software environment for integrated models of biomolecular interaction networks. Genome Res..

[26] Bader G.D., Hogue C.W. (2003). An automated method for finding molecular complexes in large protein interaction networks. BMC Bioinform..

[27] Zhou G., Soufan O., Ewald J., Hancock R.E.W., Basu N., Xia J. (2019). NetworkAnalyst 3.0: A visual analytics platform for comprehensive gene expression profiling and meta-analysis. Nucleic Acids Res..

[28] Daina A., Michielin O., Zoete V. (2017). SwissADME: A free web tool to evaluate pharmacokinetics, drug-likeness and medicinal chemistry friendliness of small molecules. Sci. Rep..

[29] Banerjee P., Kemmler E., Dunkel M., Preissner R. (2024). ProTox 3.0: A webserver for the prediction of toxicity of chemicals. Nucleic Acids Res..

[30] O’Boyle N.M., Banck M., James C.A., Morley C., Vandermeersch T., Hutchison G.R. (2011). Open Babel: An open chemical toolbox. J. Cheminform..

[31] Eberhardt J., Santos-Martins D., Tillack A.F., Forli S. (2021). AutoDock Vina 1.2.0: New Docking Methods, Expanded Force Field, and Python Bindings. J. Chem. Inf. Model..

[32] UniProt C. (2025). UniProt: The Universal Protein Knowledgebase in 2025. Nucleic Acids Res..

[33] Kawai T., Ikegawa M., Ori D., Akira S. (2024). Decoding Toll-like receptors: Recent insights and perspectives in innate immunity. Immunity.

[34] Malekafzali B., Sattari M., Keyvanfar S. (2014). Correlation between salivary Toll like receptor-2 concentration and early childhood caries. Iran. J. Immunol..

[35] Elgadir T.A., El-Gamal B., Ellatif M.A., Nasif K.A., Omer S., Mohieldeen M., Patel A.A., Amanullah M., Malik A., Mahfouz A.A. (2024). In-silico and in-vitro studies revealed alpha-amyrin as a potent pnhibitor of TLR2 for the therapeutics of bacterial infection and sepsis. Cell Mol. Biol..

[36] Mookherjee N., Lippert D.N., Hamill P., Falsafi R., Nijnik A., Kindrachuk J., Pistolic J., Gardy J., Miri P., Naseer M. (2009). Intracellular receptor for human host defense peptide LL-37 in monocytes. J. Immunol..

[37] Gyll J., Ridell K., Ohlund I., Karlsland Akeson P., Johansson I., Lif Holgerson P. (2018). Vitamin D status and dental caries in healthy Swedish children. Nutr. J..

[38] Gomez-Garcia A.P., Lopez-Vidal Y., Pinto-Cardoso S., Aguirre-Garcia M.M. (2022). Overexpression of proinflammatory cytokines in dental pulp tissue and distinct bacterial microbiota in carious teeth of Mexican Individuals. Front. Cell. Infect. Microbiol..

[39] Kovacs C.J., Faustoferri R.C., Bischer A.P., Quivey R.G. (2019). *Streptococcus mutans* requires mature rhamnose-glucose polysaccharides for proper pathophysiology, morphogenesis and cellular division. Mol. Microbiol..

[40] Yamashita Y., Shibata Y., Nakano Y., Tsuda H., Kido N., Ohta M., Koga T. (1999). A novel gene required for rhamnose-glucose polysaccharide synthesis in *Streptococcus mutans*. J. Bacteriol..

[41] van der Beek S.L., Zorzoli A., Canak E., Chapman R.N., Lucas K., Meyer B.H., Evangelopoulos D., de Carvalho L.P.S., Boons G.J., Dorfmueller H.C. (2019). Streptococcal dTDP-L-rhamnose biosynthesis enzymes: Functional characterization and lead compound identification. Mol. Microbiol..

[42] Atta L., Mushtaq M., Siddiqui A.R., Khalid A., Ul-Haq Z. (2024). Targeting glucosyltransferases to combat dental caries: Current perspectives and future prospects. Int. J. Biol. Macromol..

[43] Atta L., Siddiqui A.R., Mushtaq M., Munsif S., Nur E.A.M., Ahmed A., Ul-Haq Z. (2025). Molecular insights into antibiofilm inhibitors of *Streptococcus mutans* glucosyltransferases through in silico approaches. Sci. Rep..

[44] Wong F., Krishnan A., Zheng E.J., Stark H., Manson A.L., Earl A.M., Jaakkola T., Collins J.J. (2022). Benchmarking AlphaFold-enabled molecular docking predictions for antibiotic discovery. Mol. Syst. Biol..

[45] Scardino V., Di Filippo J.I., Cavasotto C.N. (2023). How good are AlphaFold models for docking-based virtual screening?. iScience.

[46] De Vivo M., Masetti M., Bottegoni G., Cavalli A. (2016). Role of Molecular Dynamics and Related Methods in Drug Discovery. J. Med. Chem..

[47] Daoud N.E., Borah P., Deb P.K., Venugopala K.N., Hourani W., Alzweiri M., Bardaweel S.K., Tiwari V. (2021). ADMET Profiling in Drug Discovery and Development: Perspectives of In Silico, In Vitro and Integrated Approaches. Curr. Drug Metab..

[48] Venkataraman M., Rao G.C., Madavareddi J.K., Maddi S.R. (2025). Leveraging machine learning models in evaluating ADMET properties for drug discovery and development. ADMET DMPK.

